# Mental Health in Anesthesiology and ICU Staff: Sense of Coherence Matters

**DOI:** 10.3389/fpsyt.2018.00440

**Published:** 2018-09-19

**Authors:** Sarah K. Schäfer, Johanna Lass-Hennemann, Heinrich Groesdonk, Thomas Volk, Hagen Bomberg, Marlene Staginnus, Alexandra H. Brückner, Elena Holz, Tanja Michael

**Affiliations:** ^1^Division of Clinical Psychology and Psychotherapy, Department of Psychology, Saarland University, Saarbrücken, Germany; ^2^Department of Anesthesiology and Intensive Care, Saarland University Medical Center, Homburg, Germany

**Keywords:** resilience, stress, hospital staff, intensive care, post-traumatic stress, PTSD, locus of control, sense of coherence

## Abstract

**Background:** Hospitals, and particularly intensive care units (ICUs), are demanding and stressful workplaces. Physicians and nurse staff are exposed to various stressors: emergency situations, patients' deaths, and team conflicts. Correspondingly, several studies describe increased rates of PTSD symptoms and other mental health problems in hospital staff. Therefore, it is important to identify factors that lower the risk of psychopathological symptoms. High levels of sense of coherence (SOC) and general resilience as well as an internal locus of control (LOC) have already been identified as important health-benefitting factors in medical staff. The current study aimed to evaluate their unique impact in an ICU and an anesthesiology unit.

**Method:** The cross-sectional online survey investigated SOC, LOC, general resilience, general mental health problems as well as PTSD symptoms in nurses and physicians within an ICU and an anesthesiology unit (*N* = 52, 65.4% female). General mental health problems were assessed using the ICD-10-Symptom-Rating (ISR) and PTSD symptoms were measured using the PTSD Checklist for DSM-5 (PCL-5). The Sense of Coherence Scale (SOC-L9) assessed SOC, the Resilience Scale (RS-11) measured general resilience, and LOC was determined using a 4-item scale for the assessment of control beliefs (IE-4).

**Results:** As expected, SOC, *r* = −0.72, *p* < 0.001, general resilience, *r* = −0.46, *p* < 0.001, and internal LOC, *r* = −0.51, *p* < 0.001, were negatively correlated with general mental health problems while an external LOC showed a positive association, *r* = 0.35, *p* = 0.010. However, in a multiple regression model, *R*^2^ = 53.9%, *F*_(4, 47)_ = 13.73, *p* < 0.001, only SOC significantly predicted general mental health problems by uniquely accounting for 13% of the variance. For PTSD symptoms, which were highly correlated with general mental health problems, a similar pattern of results was found.

**Conclusion:** SOC was found to be the most important correlate of both general mental health problems and PTSD symptoms in an ICU and an anesthesiology unit. Thus, if further evidenced by longitudinal studies, implementing interventions focusing on an enhancement of SOC in training programs for ICU and anesthesiology unit staff might be a promising approach to prevent or reduce psychopathological symptoms.

## Introduction

Hospitals are highly demanding and stressful workplaces. Often, patients and their relatives find themselves in unsettling situations and the medical personnel need to respond appropriately and quickly to their medical needs. Further, hospital staff are exposed to various stressors including medical emergency situations, patients' deaths, time pressure, steep hierarchies, and team conflicts. Particularly in intensive or critical care units (ICUs), even minor errors could have fatal consequences, including cases of death. At the same time, the working conditions in hospitals in many countries are far from optimal: wards are understaffed (1–3), shiftwork is common, shifts tend to be too long (4), and especially nurses receive only little recognition for their demanding work (5).

Previous research shows that these poor and stressful working conditions are linked to relatively high rates of burnout and other symptoms of mental distress (6–10). Stress-related symptoms are especially present in ICU staff (11–14). For instance, a survey in the UK found that 12% of ICU physicians, compared to 5% of the general population (15), reported clinically relevant depressive symptoms and 3% were bothered by suicidal thoughts (16). Apart from depression, secondary traumatization represents a further potential consequence of working in an ICU: 18% of the nurse staff in a university hospital in the United States not only meet the criteria of burnout syndrome, but also exceed the cut-off criteria for post-traumatic stress disorder (PTSD) (17). The study further reports that particularly ICU nurses are additionally burdened by work-related nightmares. In line with the described findings, Domiguez-Gomez and Rutledge (18) find high rates of PTSD core symptoms (intrusion, avoidance, and arousal) in emergency nurses. Overall, the high prevalence of stress-related symptoms seems to be the consequence of ongoing exposure to unpredictable stressful events at work and difficulties in employing coping strategies. The occurrence of psychopathological symptoms is critical in two ways: Firstly, hospital personnel and especially ICU staff members are at serious risk of developing mental disorders. The presence of psychopathological problems further impedes the ability of these individuals to cope with their work-related stressors and aversive experiences and might also have a negative impact on the management of private stressors. Secondly, several studies describe that these problems subsequently impair the provided quality of care (3, 19, 20).

However, even though hospitals and especially ICUs are stressful workplaces, not all nurses and physicians respond equally to those strains. Notably, a substantial proportion of hospital staff is able to successfully deal with their demanding work environment over periods of many years (21). Given these differences, it seems crucial to identify factors relevant to successful coping processes at highly stressful workplaces. In this context one of the frequently discussed concepts is Aaron Antonovsky's theory of salutogenesis (22). Contrary to other theories, which mainly center on the development of (psycho-)pathology, the salutogenesis model conceptualizes health as being at one end of a continuum from *ease* (absolute health) to *dis-ease* (absolute illness). In this regard, sense of coherence (SOC) describes a global orientation of confidence in one's ability to cope with and overcome stressful and challenging situations in life (23). SOC enables individuals to manage stressful experiences by mobilizing their internal as well as external resources to cope with specific problems and situations. Additionally, a strong SOC also comprises a feeling of meaningfulness that provides the individual with the belief that the demands and challenges of life are worth facing. Thereby, it allows individuals to move toward the *ease* end of the described continuum. Within the salutogenesis framework, SOC is described as a dispositional orientation, shaped by early life experiences between the ages of 0 and 30, rather than a state variable (24). This view was supported in longitudinal studies (25, 26), but see (27). In the context of stressful working environments, individuals who have developed a higher level of SOC should be more successful in dealing with stressors and demands and thus be more likely to maintain their mental health. Several studies already show that higher levels of SOC are associated with less stress-related and depressive symptoms in hospital staff (28–30) and paramedics (31).

However, SOC is not the only concept considered to be important in maintaining mental health in a challenging work environment. Another important—but partly overlapping—concept is general resilience. Resilience is a multidimensional construct that is defined as “the ability to adapt successfully in the face of adversity, trauma, tragedy or significant threat” ((32), p. 119). There is a continuing debate whether resilience is a personality trait or whether it develops in consequence of adversity (33): Conceptualizing resilience as a personality trait implies understanding it as a variable that inoculates individuals against the negative impact of aversive experiences. Its trait-based definition has the greatest overlap with SOC. In contrast, outcome focused approaches define resilience as a beneficial behavioral outcome in spite of aversive experiences. From this theoretical perspective, resilience may be the consequence of higher SOC levels. The conceptually wider process-based approaches understand resilience as an active process of recovering from aversive life events. With respect to this definition, SOC levels may modulate the recovery process. This heterogeneous conceptualization of resilience limits the comparability of research findings. However, several studies, which conceptualize resilience as trait, show a relationship between higher levels of resilience and less psychopathological symptoms in hospital staff (11, 21, 34).

One aspect of both SOC and resilience is locus of control (LOC), which is defined as the extent to which individuals feel they can control events in their environment (35). Several studies found that LOC is a unique concept that is important in dealing with aversive and traumatic experiences (36, 37). A stronger internal LOC is beneficial, while a more external LOC represents a risk factor for the development of psychopathological symptoms (36, 38).

**Figure 1:**
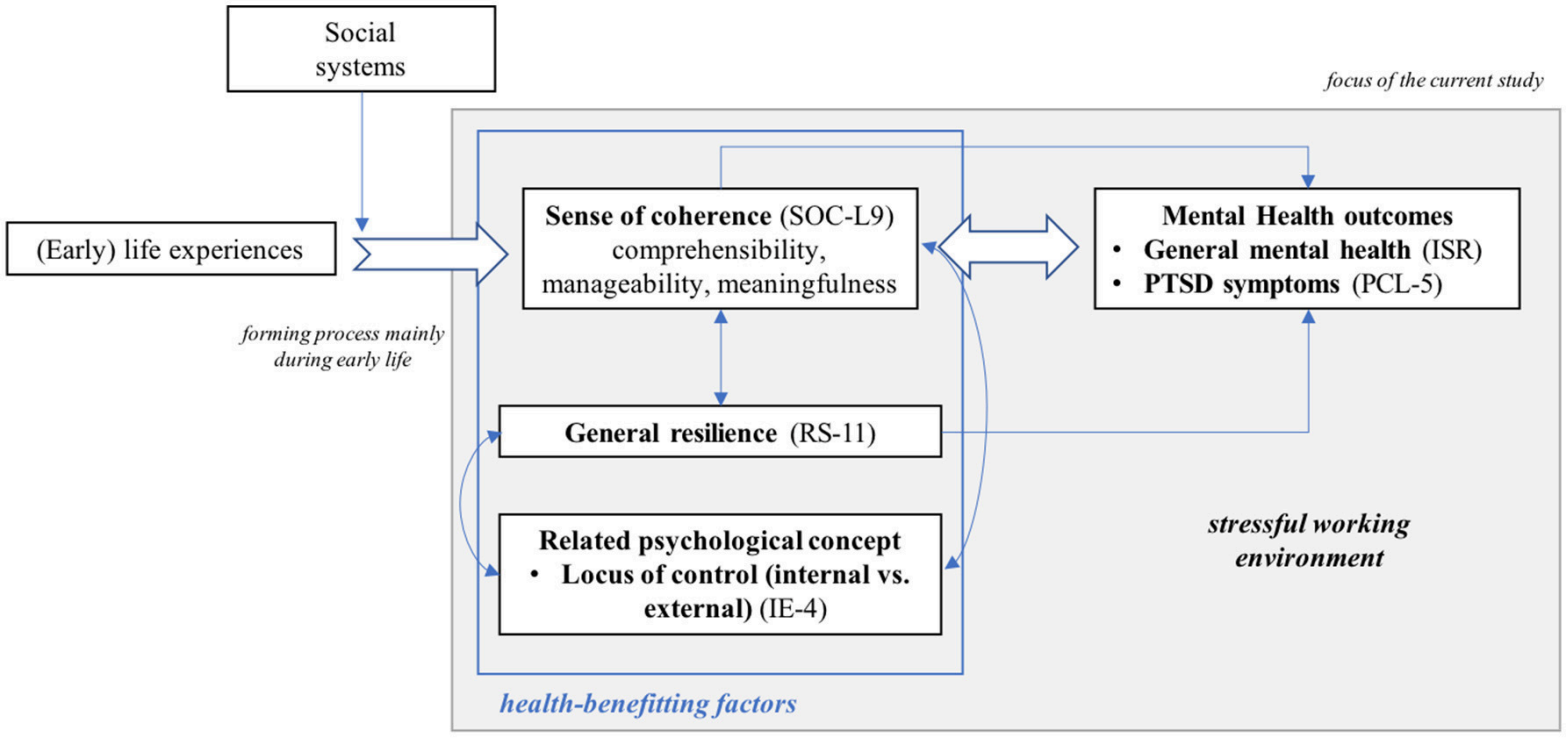
Schematic illustration of the theoretical framework.

When contrasting these health-benefitting factors with each other, SOC can be described as a persistent global orientation mainly shaped during early years of life that allows individuals to move toward the end of *ease* by making use of their resources (39). Resilience (as an outcome), however, is the ability to bounce back after an aversive event. Lastly, LOC represents a core component of both SOC and resilience. As displayed in Figure 1, SOC might function as the central and underlying orientation that contributes to the development of resilience by recruiting internal and external resources (40) and that might simultaneously be reflected in a stronger internal and a weaker external LOC, at least in Western cultures (41). Thus, upon exposure to a stressful life event, SOC, general resilience, and LOC, should be linked to psychopathological symptoms by influencing both an individual's way of behavioral coping with stressful situations and cognitive appraisal processes. In line with these assumptions, several studies conducted in hospital settings show that all three concepts are related to psychopathological symptom severity (11, 29, 30, 42). However, to the best of our knowledge, all of these studies only investigated the impact of one or two aspects, SOC *or* resilience *or* LOC, on psychopathological symptoms and thereby fail to identify the unique influence of each concept. Thus, the current cross-sectional study aims to simultaneously investigate the impact of SOC, general resilience, and LOC on general mental health problems and stress-related symptoms in an ICU and an anesthesiology unit.

Based on previous findings that describe SOC as the strongest correlate of psychopathology symptoms compared to other variables (31, 43) and our theoretical framework, we expect SOC to be the strongest correlate of current general mental health problems and post-traumatic stress in an ICU and an anesthesiology unit. General resilience, which some research suggests results from a well-developed SOC, should show a positive relationship with SOC while being negatively correlated with respondents' current symptom burden. The same pattern of correlations should also emerge for an internal LOC while a stronger external LOC should be positively linked to general mental health problems and post-traumatic stress. However, in a model containing all variables, we expect SOC as the central and most comprehensive concept to show the strongest association with the current symptom burden, and general resilience as well as an internal or external LOC to have a considerably lower unique influence.

## Materials and methods

### Sample recruitment

The study sample was recruited at Saarland University Medical Center in Homburg, Germany. Medical staff of an ICU and an anesthesiology unit were asked to take part in the study via e-mail and by handing out flyers during team meetings. The respondents did not receive any payment for their participation. While the study was undertaken, approximately 100 nurses and physicians were employed in the two units. The study was reviewed and approved by the ethics committee of Saarland University (16-2). The survey was administered as an online version using SoSci Survey (44) and as paper and pencil version with a prepaid return envelope. The participation rate was 52.0% (*N* = 52), 47 respondents used the online version, five questionnaires were received by mail.

### Sample characteristics

Thirty-four women (65.4%) and 18 men (34.6%) participated in the study. This gender difference reflects the gender imbalance amongst the medical staff of both units. The mean age was 39 years (*SD* = 10 years, range: 23–57 years).

Twenty-seven respondents were part of the nursing team (70.4% worked full-time), while 25 worked as physicians (72.0% worked full-time). The average job experience was 14 years (*SD* = 11 years, range: 1–37 years). All respondents carried out shift work (night shifts included). The nursing team members reported a mean working time of 34.71 h per week while the physicians reported working 35.33 h a week.

### Measures

*Socio-demographic and occupational information*. The questionnaire began with 20 questions on socio-demographic and occupational information (e.g., age, sex, working hours per week, work experience). Subsequently, a set of questionnaires on psychopathological symptoms and health-benefitting factors followed.

#### Health-benefitting factors

*Sense of coherence*. SOC was assessed using a German short version of the SOC scale developed by Antonvosky (SOC-L9 (45), English original scale (46)). SOC-L9 measures SOC using nine items which are rated on a bipolar seven-point scale. Cronbach's alpha (α) was 0.81 and item-total correlations ranged between *r* = 0.40 and *r* = 0.60 supporting the reported good psychometric quality of the short scale.

*General Resilience*. The Resilience Scale 11 (RS-11 (47), English original (48)) assesses individual general resilience when an individual is faced with a stressful life event. RS-11 was developed as short version of the Resilience Scale 25. The current study employed the German short version (RS-11); its reliability was satisfying with a Cronbach's α of 0.89. The item-total correlations ranged between *r* = 0.28 and *r* = 0.72.

*Locus of control*. Internal and external locus of control were assessed using a brief scale for the assessment of internal and external control beliefs (IE-4 (49)). The instrument consists of two subscales measuring perceived internal and external control, each containing two items. All items are rated on a five-point scale. Items of both scales correlated as expected, *r*_*internal*_ = 0.48, *p* < 0.001, and *r*_*external*_ = 0.58, *p* < 0.001. The two scales were moderately negatively correlated, *r* = −0.38, *p* = 0.006.

#### Post-traumatic stress and psychopathological symptoms

*ICD 10 symptom rating*. Mental health was assessed using the ICD 10 symptom rating (ISR (50)). All ISR items are derived from the ICD 10 diagnostic criteria and assess the symptoms of mental health difficulties on five syndrome subscales with three to four items each. An additional sixth scale covers 12 symptoms (one item each) which may occur within different syndromes. The individual items can also be taken as a first indication of a specific disorder, such as PTSD. All items are rated on a five-point scale. The total score can be used as an indicator of an individual's overall mental health problems (51). Psychometric qualities of the scale have been described as satisfactory (52). In the current study, its good internal consistency was reflected in an overall Cronbach's α of 0.86 for the total score.

*Post-traumatic stress symptoms*. Post-traumatic stress symptoms were assessed using the German version of the PTSD Checklist (PCL-5 (53); English original: 54) which relies on the diagnostic criteria of DSM-5 (55). The PCL-5 measures PTSD symptoms based on 20 items which are rated on a five-point scale. In the current sample, the PCL demonstrated excellent reliability reflected in a Cronbach's α of 0.91. Item-total correlations ranged between 0.39 and 0.74. According to Krüger-Gottschalk et al. (53) a total score of 33 can be used as cutoff criterion for a provisional PTSD diagnosis.

### Data analyses

Data collection was performed using SoSci Survey (44) or a paper and pencil version of the questionnaire. The paper questionnaires were manually entered using the online version of the questionnaire. All relevant data analyses were conducted using SPSS version 24 (56) with the exception of the reported path analyses which were carried out with SPSS Amos version 24 (57). Descriptive statistics included the computation of means, standard deviations, and frequencies. Missing data was < 5% on the relevant scales and was replaced using scale means per subject.

To assess differences between nursing staff and physicians, MANOVAs and *t*-tests for independent samples were conducted on an exploratory basis. Bonferroni-Holm's correction was applied to control for the influence of multiple testing (58). Pearson bivariate correlation coefficients were used to characterize the relationship between general resilience, SOC, LOC, general mental health problems and post-traumatic stress symptoms. To determine to what extent the significant bivariate variables uniquely predict general mental health problems and post-traumatic stress symptom severity, multiple regression models were calculated. The regression residuals were normally distributed, and predictors were not multicollinear. To assess the unique amount of variance accounted for by one predictor, we calculated hierarchical regressions including all variables in the last step. The change in *R*^2^ (Δ*R*^2^) reflects the unique amount of variance explained by the variable included in the last step. The significance of Δ*R*^2^ can be assessed by Δ*F*. Based on our theoretical assumptions and current findings from the multiple regression analyses, path analyses were conducted to visualize our findings and to test a mediating hypothesis on an exploratory basis. Parameters were attained by maximum likelihood estimations and multivariate normality was indicated by Mardia's test. Model fit was assessed based on χ^2^-values (α was set to 0.20 to control for type II errors). Since the small study sample might reduce the power of the χ^2^-test, other fit indices were used to assess the model fit. Specifically, Root Mean Square Error Approximation (RMSEA), Normal Fit Index (NFI), and Tucker-Lewis Index (TLI) were evaluated. A RMSEA below 0.06 as well as a NFI and a TLI ≥0.95 can be seen as indicators of well-fitted models (59).

## Results

### Psychopathological symptoms

#### General mental health problems

With respect to general mental health problems as reflected in the ISR total score, only nursing staff showed a significantly increased symptom burden compared to the cut-off score, *t*_(26)_ = 3.96, *p* = 0.001. Particularly depression, *t*_(26)_ = 4.30, *p* < 0.001, and eating disorder symptoms, *t*_(26)_ = 4.01, *p* < 0.001, were significantly higher than the cut-off (for a detailed overview, see Table 1). Differences between nurses and physicians were examined using the between-group effect in a MANOVA. After applying Bonferroni-Holm's correction to all tests, there were no significant between-group differences on any of the ISR scales. However, in terms of the multivariate profile, nurses showed a significantly higher symptom burden, *F*_(6, 45)_ = 2.70, *p* = 0.025, η^2^ = 0.27, which was also reflected in a significantly higher ISR total score, *t*_(50)_ = −3.15, *p* = 0.003, η^2^ = 0.17.

**Table 1:** Mean scores and standard deviations for general mental health problems in nurses and physicians.

				**Nurses vs. physicians**		
	**Physicians (*n* = 25)**	**Nursing staff (*n* = 27)**	**Total**	***F*_(1, 50)_**	***p***	**$\eta_{p}^{2}$**
Depression	1.09 (0.95)	1.51 (0.93)^*^	1.31 (0.95)	2.66	0.109	0.05
Anxiety	0.67 (0.83)	0.87 (0.78)	0.76 (0.80)	0.84	0.365	0.02
Obsessive-compulsive	0.27 (0.62)	0.47 (0.65)	0.37 (0.64)	1.31	0.257	0.03
Somatic symptoms	0.08 (0.20)	0.38 (0.71)	0.24 (0.55)	4.25	0.044	0.08
Eating disorders	0.35 (0.59)	0.93 (0.78)^*^	0.65 (0.75)	9.05	0.004	0.15
Additional scale	0.35 (0.31)	0.60 (0.42)	0.48 (0.39)	5.66	0.021	0.10
Total score	0.45 (0.37)	0.77 (0.35)	0.62 (0.39)			

Statistically significant results compared to the ISR cut-off scores are marked

* *if p < 0.05 after applying Bonferroni-Holm's correction. F statistics, p-values and $\eta_{p}^{2}$ relate to the between-group comparison of physicians and nursing staff*.

#### Post-traumatic stress symptoms

Descriptive statistics for PTSD symptoms are shown in Table 2. The nursing staff showed significantly higher scores than the physicians as indicated by the MANOVA between-group effect, *F*_(4, 47)_ = 2.77, *p* = 0.038, η^2^ = 0.19, and the PCL total score, *t*_(50)_ = −3.08, *p* = 0.003, η^2^ = 0.15. Furthermore, regarding the subscales, nurses showed a significantly higher symptom burden only on the avoidance subscale. Based on a cut-off score of 33 (60), five cases were clinically relevant from which three were members of the nursing team.

**Table 2:** Mean scores and standard deviations for the PCL-5 scales and the total score.

	**Intrusion**	**Avoidance**	**Negative alterations in cognition and mood**	**Alterations in arousal and reactivity**	**Total sore**
Physicians (*n* = 25)	1.60 (2.75)	1.24 (1.98)	3.44 (3.66)	2.88 (3.46)	9.16 (9.72)
Nursing staff (*n* = 27)	3.76 (4.09)	3.74 (3.95)	5.38 (5.38)	5.70 (4.31)	18.30 (11.50)
Total	2.72 (3.65)	2.54 (3.38)	4.62 (4.14)	4.03 (3.59)	13.90 (11.54)
*F*_(1, 50)_	4.90	8.12	4.13	5.35	
*p*	0.031	0.006^*^	0.048	0.025	
$\eta_{p}^{2}$	0.09	0.14	0.08	0.10	

Statistically significant results are marked

* *if p < 0.05 after applying Bonferroni-Holm's correction. F statistics, p-values and $\eta_{p}^{2}$ relate to the between-group comparison of physicians and nursing staff*.

### Health-benefitting factors

Table 3 shows the descriptive statistics for the RS-11, SOC-L9, and IE-4. There were no differences with respect to resilience, nor SOC or LOC between physicians and nursing staff after applying the Bonferroni-Holm's correction. Moreover, no significant between-group differences were found in a MANOVA including all measures, *F*_(4, 47)_ = 1.18, *p* = 0.333, η^2^ = 0.09.

**Table 3:** Mean scores and standard deviations for resilience, SOC and LOC.

	**Resilience (RS-11)**	**Sense of Coherence (SOC-L9)**	**Locus of control—internal (IE-4)**	**Locus of control—external (IE-4)**
Physicians (*n* = 25)	58.76 (12.16)	47.32 (7.96)	4.04 (0.56)	2.30 (0.74)
Nursing staff (*n* = 27)	59.17 (10.62)	43.19 (9.63)	3.83 (0.88)	2.43 (0.93)
Total	58.97 (11.29)	45.18 (9.02)	3.93 (0.75)	2.37 (0.83)
*F*_(1, 50)_	0.02	2.82	0.99	0.29
*p*	0.896	0.099	0.324	0.592
$\eta_{p}^{2}$	0.00	0.05	0.02	0.01

*F statistics, p-values and η_p_2 relate to the between-group comparison of physicians and nursing staff*.

### Bivariate correlations

Bivariate correlations between health-benefitting factors and general mental health problems and post-traumatic stress symptoms are displayed in Table 4. SOC, resilience, and internal and external LOC were significantly correlated with both general mental health problems as measured by the ISR total score and PTSD symptoms as assessed by the PCL-5 total score.

**Table 4:** Bivariate correlations of all relevant variables.

	**1**	**2**	**3**	**4**	**5**	**6**
ISR total score (1)	*0.86*					
PCL total score (2)	0.78^**^	*0.91*				
Sense of coherence (3)	−0.72^**^	−0.62^**^	*0.81*			
Resilience (4)	−0.46^**^	−0.33^*^	0.52^**^	*0.89*		
Locus of control—internal (5)	−0.51^**^	−0.47^**^	0.58^**^	0.28^*^	*0.48*	
Locus of control—external (6)	0.35^*^	0.44^**^	−0.54^**^	−0.07	−0.38^**^	*0.58*

* *p < 0.05*,

** *p < 0.01. The italicized diagonal contains Cronbach's Alpha coefficients as a measure of internal consistency*.

### Regression models

Regression models were calculated for general mental health problems and post-traumatic stress symptoms. Table 5 displays the regression model of general mental health problems. The overall model predicted 53.9% of the variance (adjusted *R*^2^ = 50.0%) of general mental health problems, *F*_(4, 47)_ = 13.73, *p* < 0.001. However, by accounting for 13.4% of the variance, only SOC was found to be a significant predictor of mental health problems, *t*_(47)_ = −3.70, *p* < 0.001. In comparison to the significant bivariate correlations (see Table 4), the regression weights for resilience, β = −0.12, *t*_(47)_ = −1.00, *p* = 0.323, internal LOC, β = −0.15, *t*_(47)_ = −1.21, *p* = 0.234, and external LOC, β = −0.02, *t*_(47)_ = −0.17, *p* = 0.856, were strongly decreased. The regression model for PTSD symptoms is also shown in Table 5. The overall model significantly predicted PTSD symptoms, *F*_(4, 47)_ = 8.29, *p* < 0.001. Together, the variables SOC, resilience, and perceived internal and external control (LOC) accounted for 41.4% of the variance (adjusted *R*^2^ = 36.4%). However, again only SOC significantly predicted a unique amount of variance in symptom severity, Δ*R*^2^ = 0.07, *t*_(47)_ = −2.30, *p* = 0.026. As for general mental health problems, the regression weights for resilience, β = −0.07, *t*_(47)_ = −0.48, *p* = 0.637, internal LOC, β = −0.16, *t*_(47)_ = −1.15, *p* = 0.255, and external LOC, β = 0.15, *t*_(47)_ = 1.11, *p* = 0.273, were substantially lower compared to their bivariate correlations with PTSD symptoms (see Table 4).

**Table 5:** Multiple regression analysis of general mental health problems (ISR total score) and PTSD symptom severity (PCL total score).

	***B***	**SE *B***	**β**	***t***	***p***	**Δ*R^2^***	**Δ*F***
**GENERAL MENTAL HEALTH (ISR TOTAL SCORE)**							
Sense of coherence	−0.03	0.01	−0.58	−3.70	< 0.001^**^	0.13	13.68
Resilience	0.00	0.00	−0.12	−1.00	0.323	0.01	1.00
Locus of control—internal	−0.08	0.06	−0.15	−1.21	0.234	0.01	1.45
Locus of control—external	0.01	0.06	−0.02	−0.17	0.865	0.00	0.03
**PTSD SYMPTOMS (PCL TOTAL SCORE)**							
Sense of coherence	−0.52	0.23	−0.41	−2.30	0.026^*^	0.07	5.29
Resilience	−0.07	0.14	−0.07	−0.48	0.637	0.00	0.22
Locus of control—internal	−2.45	2.13	−0.16	−1.15	0.255	0.02	1.33
Locus of control—external	2.13	1.92	0.15	1.11	0.273	0.02	1.23

* *p < 0.05*;

** *p < 0.01. The columns reporting ΔR^2^ and ΔF refer to hierarchical regression analyses in which each variable was included in the last step. p-values of the beta-weights and ΔF are equal and hence not reported twice*.

### Path analyses

**Figure 2:**
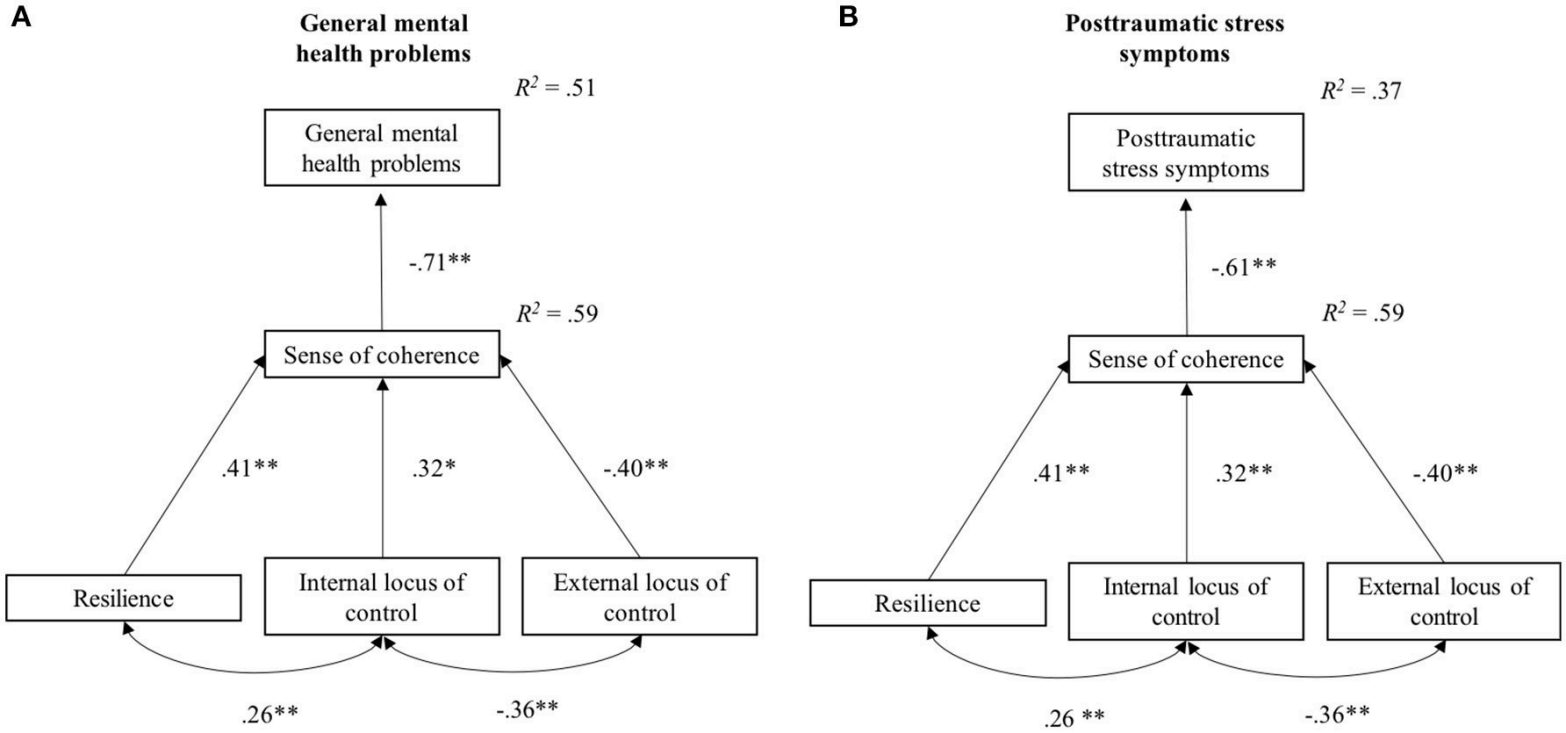
Path models for general mental health problems **(A)** and post-traumatic stress symptoms **(B)** with standardized regression weights, correlations, and explained variances. ^*^p < 0.05, ^**^p < 0.01.

Based on the theoretical framework and the findings from the regression analyses, two path models were established to explain general mental health problems and severity of post-traumatic stress symptoms based on SOC and resilience, internal LOC, and external LOC as health-benefitting factors. Figure 2 shows both path models including standardized regression weights as well as correlations between internal and external LOC and between resilience and internal LOC.

The established model for general mental health problems explained 50.5% of the variance in symptom severity. Moreover, resilience, β = 0.41, *p* < 0.001, internal LOC, β = 0.32, *p* = 0.001, as well as external LOC, β = −0.40, *p* < 0.001, accounted for 59.0% of the variance in SOC. For the model of general mental health problems, the covariance matrix predicted by the model did not significantly differ from the observed covariance matrix, χ^2^_(4)_ = 2.96, *p* = 0.564. The Mardia's test remained non-significant, *C.R*. = 1.72 < 1.96, indicating multivariate normal distribution. RMSEA was 0.00 (*p*_CLOSE_ = 0.624), the NFI was 0.97, and TLI was 1.03. All estimated direct effects were significant. Additionally, as displayed in Table 6 the indirect effects of resilience, internal LOC, and external LOC, on general mental health problems were significant and thus mediated by SOC. With respect to symptoms of post-traumatic stress, the model accounted for 37.1% of the variance in symptom severity. Furthermore, the covariance matrix implied by the model and the observed matrix did also not differ significantly, χ^2^_(4)_ = 3.20, *p* = 0.525. There was no evidence of a violation of multivariate normality in the Mardia's test, *C.R*. = 1.87 < 1.96. The fit indices showed a well-fitting model, RMSEA = 0.00 (*p*_CLOSE_ = 0.587), NFI = 0.96, TLI = 1.03. Moreover, the indirect effects of resilience, internal LOC, and external LOC mediated by SOC were significant (see Table 6).

**Table 6:** Indirect effects (β*)* of general resilience and LOC on general mental health problems and post-traumatic stress symptoms.

	**Resilience**	**LOC internal**	**LOC external**
General mental health	−0.29^*^	−0.23^*^	0.28^*^
CIs from bootstrapping	−0.42 (−0.16)	−0.40 (−0.05)	0.16 (−0.43)
Post-traumatic stress symptoms	−0.25^*^	−0.20^*^	0.24^*^
CIs from bootstrapping	−0.38 (−0.15)	−0.37 (−0.05)	0.12 (−0.41)

* *p < 0.05. Significance of indirect effects was assessed using Bootstrapping based on 2,000 samples and bias-corrected 95% confidence intervals (CI)*.

## Discussion

In the current study, SOC emerged as the most important predictor for general mental health problems as well as post-traumatic stress symptoms in an ICU and an anesthesiology unit. In regression models based on SOC, general resilience, and LOC, 53.9% of the variance in general mental health problems and 41.4% of the variance in post-traumatic stress symptoms were explained. General resilience as well as internal and external LOC were significantly correlated with both general mental health problems and post-traumatic stress symptoms but did not explain unique variance in the regression models when SOC was included. In comparison to the bivariate correlations the regression weights of general resilience and internal and external LOC were substantially lower in both models.

These results were further visualized in path analyses that also showed that SOC was the most important predictor of general mental health problems and post-traumatic stress symptoms, rendering the inclusion of direct paths from general resilience and LOC components redundant with regard to both symptom measures. However, general resilience as well as internal and external LOC had significant indirect effects on symptom severity measures mediated by SOC on symptom severity measures.

The current findings are in line with previous research identifying SOC as an important factor in dealing with work-related stress in hospitals (29, 30, 61). For instance, a study in nursing students demonstrated a moderating effect of SOC on the association between post-traumatic stress and quality of life (28): only nursing students with low levels of SOC showed a negative association between stress and quality of life; for nursing students with medium and high levels of SOC no association emerged. Individuals with higher levels of SOC may be more efficient in dealing with aversive experiences and high levels of stress in everyday occupational life. In order to establish the temporal relationship between SOC and the development of psychopathological symptoms, longitudinal studies need to be conducted. If such studies support SOC's role as a crucial factor in the development and course of mental health problems, further longitudinal studies should establish by which (cognitive and behavioral) mechanisms SOC influences the development of psychopathological symptoms. Based on the salutogenesis theory it is plausible to assume that SOC as a global orientation may influence the perception of stressors as well as the use of (dys-)functional coping strategies. A recent qualitative study on highly resilient nurses identified three main factors for functional coping with occupational stressors (62): the presence of work-related and external support networks, individual factors, such as self-care and self-motivation, and the ability to organize work in a way that supports one's personal sense of fulfillment. One can assume that all these factors are positively related with SOC, as SOC enables individuals to mobilize different resources and to believe in their ability to effectively change problematical aspects of their work places. A stronger SOC might also allow individuals to experience their work as more meaningful, and thus, more satisfying. Thereby, high levels of SOC might prevent feelings of helplessness and unpredictability, which are highly related to perceived stress and, in turn, the development of psychopathological symptoms (63).

Even though SOC, general resilience and LOC were all significantly correlated with mental health outcomes, only SOC explained unique variance in the regression models. Correspondingly, a previous study in paramedics identified SOC, but not general resilience, as a significant predictor of PTSD symptom severity (31). A further, larger study in a student sample found SOC to have incremental validity above general resilience and other health-benefitting factors (i.e., optimism, self-compassion) in predicting psychological distress (43). However, the current findings should not be taken to indicate that general resilience and LOC are irrelevant. Instead, the results underline the large conceptual overlap between the investigated concepts, rendering general resilience and LOC redundant in complex models. On a conceptual level, one might argue that high levels of SOC might be a predisposition formed during early life that results in high levels of general resilience and a rather internal LOC. From this perspective, the latter two might be understood as beneficial outcomes of an advantageous SOC development.

With respect to group differences between nurses and physicians, the nursing staff showed an overall higher symptom burden, which is consistent with previous studies in hospitals (9) and a recent study conducted in an ICU and an anesthesiology unit (64). Interestingly, no group differences were found between nurses and physicians regarding SOC, general resilience, and LOC. Due to the cross-sectional nature of the data, it is not clear if the considerable higher (stress-related) symptom burden in the nursing subsample might reflect a short-term stress situation in the nursing staff or if it hints at the presence of constantly higher levels of persistent work-related stressors in this group.

### Limitations

The present study has several limitations: Firstly, the relatively small sample of 52 nurses and physicians limits the findings' representativeness. Moreover, it is plausible to assume that highly stressed staff members decided not to participate in the current study. Unfortunately, it was not possible to obtain any information on the staff members who did not participate in the study. Moreover, the small sample size limits the statistical power of all analyses. Specifically, in spite of the substantially lower regression weights, general resilience and LOC might have remained significant predictors of general mental health problems and PTSD symptoms in a larger sample.

Secondly, due to organizational guidelines it was not possible to differentiate between respondents working in the ICU and those who work in the anesthesiology unit.^1^ Thus, the impact of the differing demands in an ICU and an anesthesiology unit could not be addressed, but they might have influenced the results by acting as a moderating variable. However, a recent study, which compared stress levels in ICU and anesthesiology staff, did not report any differences (64).

Thirdly, the current findings show that SOC, an internal LOC and general resilience are highly correlated aspects that all show significant relationships with the current symptom burden on a bivariate level. In regression analyses, SOC remained the only significant predictor of general mental health problems and post-traumatic stress symptoms. The proposed mediator model is one potential explanation of the observed interrelationships but is—irrespective of its good fit—not the only possible interpretation, nor does the model allow for true causal conclusions. With respect to causality, the current findings seem to suggest that SOC plays an important role in the development of mental health problems in ICUs and anesthesiology units. However, the data could also be interpreted differently: For instance, it would be plausible to assume that low levels of SOC and general resilience as well as an external LOC might only reflect existing mental health problems and can be seen as correlates instead of influencing factors. Moreover, the central role of SOC could also indicate that current psychopathological symptoms affect SOC the most. Therefore, only longitudinal studies will give a greater insight into the causal and temporal development of mental health problems in ICUs and anesthesiology units as well as in hospital staff in general. Prospective research in larger samples should also include further measures of health-benefitting factors (e.g., dispositional optimism, self-efficacy or openness) and a broader assessment of health outcomes (e.g., burnout symptoms or measures of secondary traumatization).

Lastly, the current study did not have a control group of nurses and physicians not working in an ICU or an anesthesiology unit. Hence, it cannot be concluded that the reported levels of stress and the symptom burden arise from specific occupational characteristics in these units. They may reflect general occupational stress in medicine. However, our findings are in line with previous studies that reported particularly high levels of post-traumatic stress and burnout in ICU staff (13, 65).

### Implications

Given that SOC seems to be an important correlate of nurses' and physicians' mental health, hospitals should consider offering training courses, especially if the assumed relationship is further supported by longitudinal data. Although SOC has been conceptualized as a stable concept, findings in various populations have shown that it can be influenced, even in later life (66–68). Therefore, several means by which to include the salutogenic approach in professional mental health care have been proposed (69). For example, two mindfulness-based intervention programs for nurses were successful in enhancing SOC (70, 71). Based on the current findings and further evidence by longitudinal studies, it might be useful to offer SOC training courses especially for ICU and anesthesiology unit staff members. Furthermore, in the context of prevention, it might be beneficial to already include such courses during nursing or medicine school. Lastly, previous research has further found SOC to be positively influenced by the nursing managers' recognition behavior (72). Therefore, enhancing nursing staff's SOC by training nursing managers' ability to recognize and appreciate their staff's work performance might constitute a useful and easily implemented intervention. However, to date there is still a strong need for further longitudinal studies investigating the influence of SOC on the development and course of psychopathological symptoms to provide evidence-based recommendations.

### Conclusion

The current study underlines the important role of SOC as a correlate of general mental health problems and post-traumatic stress symptoms in nurses and physicians in an ICU and an anesthesiology unit. Future research needs to further clarify the causal and temporal influence of general resilience, LOC, and particularly SOC in the development and course of mental health problems in hospital staff.

## Data availability statement

The raw data supporting the conclusions of this manuscript will be made available by the authors, without undue reservation, to any qualified researcher.

## Author contributions

SS coordinated the data collection, analyzed the data, interpreted the results, and wrote the first draft of the manuscript; JL-H designed the study, interpreted the statistical results, and gave feedback during the manuscript writing process; HG, TV, and HB designed the study, supported the data collection process and interpreted the statistical findings; EH and AB designed the study, took part in the interpretation of the study results, and supported the manuscript writing; MS supported the data collection, conducted the statistical analyses together with SS and supported the manuscript writing as well as proof reading of the manuscript. TM contributed to the study design and supported data interpretation and manuscript writing. All authors read and approved the manuscript.

### Conflict of interest statement

The authors declare that the research was conducted in the absence of any commercial or financial relationships that could be construed as a potential conflict of interest.

## Footnotes

^1^Given the small total sample, the university hospital decided to exclude an item which would have allowed for the differentiation of ICU and anesthesiology staff members, since combined with other socio-demographic information, this might have limited the guaranteed anonymity of the survey.

## Abbreviations

ICU: intensive care unit
ISR: ICD-10-symptom-rating
LOC: locus of control
NFI: normal fit index
PCL-5: PTSD checklist for DSM-5
PTSD: post-traumatic stress disorder
RS-11: resilience scale 11
RMSEA: root mean square error approximation
SOC: sense of coherence
SOC-L9: sense of coherence scale—short form
TLI: Tucker-Lewis index
